# Thirtieth anniversary of the Brazilian Journal of Cardiovascular Surgery. And devising the next decades

**DOI:** 10.5935/1678-9741.20160061

**Published:** 2016

**Authors:** Walter J. Gomes

**Affiliations:** 1 Escola Paulista de Medicina, Universidade Federal de São Paulo (UNIFESP), São Paulo, SP, Brazil

In recent decades the Brazilian cardiovascular surgery has made milestones, an extraordinary evolution occupying a prominent position in the international scenario, with a significant increase in volume of surgical procedures, development of new surgical techniques and a local industry that supported this rise.

Similarly, the union of cardiovascular surgeons enabled the construction of a strong and thriving specialty society, which has served as a model for other guilds over the country by the vigor in the defense of their rights, achievements and attained leadership.

In the wake of these journeys, 30 years ago the Brazilian Journal of Cardiovascular Surgery was launched, consolidating its position as the official journal for promoting the scientific production of the Brazilian cardiovascular surgeons. With strong leadership working hard as editors, continuous developments and changes contributed to the journal reached this position, from the early conquests with the inclusion in Scielo portal allowing the electronic version in Portuguese and spread on the Internet to the inclusion and availability in the CTSNet portal, the Internet page of cardiovascular surgeons from around the world.

The indexing in Medline / Pubmed, the most important scientific database on the planet in health sciences, represented the maturity of the publication project and confidence in the evolution of quality.

But crucial decisions were made about the way to go, after long and exhaustive analysis. With globalization and the consequent need for dissemination of its scientific content, the adoption of the English language was natural and imperative to achieve this goal. Breaking the paradigm of a regional publication evolving to show up on the world stage. The change of the name to Brazilian Journal of Cardiovascular Surgery stood also for a challenge, with the scope to internationalize the journal and places it to assume a more representative position in the cardiovascular arena. To accomplish this goal, English language translation or editing service has been provided for authors who are not fluent in this idiom, assisting to prepare a publication-ready.

Just like the other periodicals, the BJCVS strive to publish significant and high-quality original scientific papers, which will serve for further interest in citation and therefore increasing the impact factor. The impact factor ranks the scientific excellence of the periodicals worldwide and all the efforts are focused to reach the quality level attained by the top publications in the field.

And the adoption of a new software of electronic submission, the Scholar One is intended to facilitate to local and overseas authors the straightforward submission of the manuscript, avoiding delays and expediting the publication.

The publication has been expanded for six issues a year, and the continuing medical education (CME) is provided in every issue, where the editor picks the article and write the related multiple choice questions.

Expanding the scope, the BJCVS also has served for publication of original works from multidisciplinary or multiprofessional production, specialties and professionals of related areas working along cardiovascular surgeons and making possible achieve the high standard of healthcare and scientific excellence.

Seven associate editors have been assigned to assist in every sub-area of the cardiovascular surgery with the review of different types of manuscripts, select reviewers, review reviews, and make the final decision to accept, reject, or revise. Moreover, a new team at the editorial office with Meryt Zanini as the Managing Editor and Camila Sáfadi taking over the position of Editorial Manager, greatly assisting and facilitating the work of the Chief Editor, the Associate Editors, and the reviewers. And all of them led by Professor Domingo Braile, the enthusiastic and tireless Chief Editor, whose hard work has enabled to reach this stage.

Rearrangements of the journal's sessions will permit to cover articles on Innovations and New Technologies and keep pari passu with the transformations the specialty has been through.

After all, several reasons make attractive to publish in BJCVS a relevant article. A journal with 30 years of tradition, indexed in PubMed / Medline which consequently allows the authors greater exposure of their paper, besides the free access on the Web to the full content of the articles and free of charge.

But BJCVS still have to climb higher levels, complete the internationalization process and get to be next to the best journals in the world.

